# Comprehensive validation of liquid-based cytology specimens for next-generation sequencing in cancer genome analysis

**DOI:** 10.1371/journal.pone.0217724

**Published:** 2019-06-14

**Authors:** Toshiaki Akahane, Tomomi Yamaguchi, Yasutaka Kato, Seiya Yokoyama, Taiji Hamada, Yukari Nishida, Michiyo Higashi, Hiroshi Nishihara, Shinsuke Suzuki, Shinichi Ueno, Akihide Tanimoto

**Affiliations:** 1 Department of Pathology, Kagoshima University Graduate School of Medical and Dental Sciences, Kagoshima, Japan; 2 Center for Human Genome and Gene Analysis, Kagoshima University Hospital, Kagoshima, Japan; 3 Department of Pathology, Laboratory of Cancer Medical Science, Hokuto Hospital, Obihiro, Japan; 4 Department Surgical Pathology, Kagoshima University Hospital, Kagoshima, Japan; 5 Department of Biology and Genetics, Laboratory of Cancer Medical Science, Hokuto Hospital, Obihiro, Japan; 6 Keio Cancer Center, Keio University School of Medicine, Tokyo, Japan; 7 Department of Clinical Cancer Research, Kagoshima University Graduate School of Medical and Dental Sciences, 890–8544 Kagoshima, Japan; 8 Kagoshima University Hospital Cancer Center, Kagoshima University Hospital, Kagoshima, Japan; Universita degli Studi di Napoli Federico II, ITALY

## Abstract

In addition to conventional cytology, liquid-based cytology (LBC) is also used for immunocytochemistry and gene analysis. However, an appropriate method to obtain high quality DNA for next-generation sequencing (NGS) using LBC specimens remains controversial. We determined the optimal conditions for fixation with an alcohol-based fixative for LBC and DNA extraction using cultured cancer cell lines and clinical specimens. The extracted DNA was processed for NGS after the DNA quality was confirmed based on the DNA concentration and degree of degradation. The optimal conditions for cultured cells to obtain high quality DNA were to fix the cells at a density of 6 × 10^3^ or 2 × 10^4^ cells/mL and to use the magnetic bead-based DNA extraction method. Even after storing the fixed cells for 90 days, DNA extracted using the above and other extraction kits, including membrane-based methods, did not undergo degradation. Furthermore, 5-year-old residual LBC samples demonstrated high DNA quality that was suitable for NGS. Furthermore, a cancer genome panel analysis was successfully performed with DNA extracted from cultured cells fixed at 6 × 10^3^ cells/mL for 90 days, and with DNA from residual LBC samples even after 1 year of storage. Residual LBC samples may be a useful source of DNA for clinical NGS to promote genome-based cancer medicine.

## Introduction

Tissue samples taken for pathological diagnosis, including fresh, frozen and formalin-fixed tissues, are often used for comprehensive cancer genome analysis. Recent technical developments, especially high-throughput sequencing, enables researchers to subject formalin-fixed paraffin-embedded (FFPE) tissues to cancer genome analysis using next generation sequencing (NGS) [1–4]. A major advantage of FFPE tissues over frozen tissue is that pathologists can observe the cancer lesions in the same region of the samples that is subjected to genome analysis to directly and accurately compare the histological findings and genomic profiles [5]. Using the appropriate tissue fixation with 10% phosphate-buffered neutral formalin, the DNA fragmentation would be minimal even after 5-year storage as paraffin blocks, and whole exome sequence has been successfully performed [6]. The utilization of FFPE tissues for genome sequencing might be less expensive than that of fresh frozen tissues, which requires a special facility such as deep freezers and liquid nitrogen tanks for the long-term storage of samples.

Because the improved performance of sequencers and related analytical apparatus can produce more detailed genome information, sufficiently high quality DNA without artificial modification by formalin [7, 8] is required for successful high-performance sequencing. Therefore, a novel fixation method that does not increase artificial modifications and is available for both pathological diagnosis and genome analysis is required. PAXgene fixative (Becton Dickinson, Franklin Lakes, NJ, USA) is a potential alternative to formalin that can be used for both histopathological and biomedical analysis, however, its use is known to reduce the quality of RNAs in old tissue archives [9].

On the other hand, clinical cytology specimens including alcohol fixed-, or air dried-smears and cell blocks can be used for immunocytochemical and biochemical analysis because the samples effectively preserves cellular morphology, proteins, and genes [10–12]. The development of liquid-based cytology (LBC) supports the availability of cytology samples for both cytopathology and genome biology. Furthermore, comprehensive genome analysis of LBC samples has been attempted recently in the fields of pathology and laboratory medicine [13, 14]. Studies have reported that DNA extracted from conventional cytological or LBC samples could be applied to NGS analysis [15–18], but these studies did not always include detailed descriptions of their fixation and DNA extraction methods. At present, therefore, an efficient and suitable method for DNA extraction to acquire high-quality of DNA for NGS from LBC samples has not been introduced.

The aims of this study is to clarify a suitable method for extracting DNA to perform NGS from LBC specimens. In particular, the fixation conditions, such as cell density and fixation time, and DNA extraction methods were investigated in cultured human cancer cell lines. Residual LBC samples stored at 4°C for up to 4 years were used to investigate the degree of DNA degradation after long-term storage. Furthermore, residual LBC samples stored at 4°C for 1 year, along with cultured cells stored for 90 days, were subjected to genome analysis using comprehensive cancer gene panels and NGS.

## Materials and methods

### Cell culture

Human pancreatic adenocarcinoma Capan-1, human thyroid follicular carcinoma FTC133, and human breast cancer MCF-7 cells were purchased from the American Type Culture Collection (ATCC, Manassas, VA, USA) and maintained in Dulbecco’s modified Eagle’s medium-F12 (Sigma Aldrich, St Louis, MO, USA) with 10% fetal bovine serum and 1mg/mL penicillin/streptomycin cocktail (Life Technologies, Grand Island, NY, USA) in a humidified incubator at 37°C and 5% CO_2_. The cultured cells were harvested at the 80 to 90% confluence, washed in phosphate-buffered saline (PBS), pelleted by centrifugation at 1,200 rpm and 4°C for 5min, and then fixed in 5 mL alcohol-based fixative for LBC (CytoRich Red solution, Becton Dickinson) at a cell density of 6 × 10^3^, 2 × 10^4^ and 1 × 10^5^ cells/mL. The cells were stored in solution for 7, 30, or 90 days at 4°C before DNA extraction was performed.

### Liquid-based cytology

After storage for 90 days at 4°C in CytoRich Red solution, the cultured cells were fixed on glass slides for Papanicolaou staining.

### DNA extraction and quality check

Four different DNA extraction kits were used in this study. The DNeasy Blood & Tissue kit (Qiagen, Hilden, Germany) and NucleoSpin Tissue XS kit (Takara Bio, Shiga, Japan), which use membrane-equipped spin columns for DNA extraction, as well as the Agencourt FormaPure DNA kit (Beckman Coulter, Brea, CA, USA) and Maxwell 16 FFPE Tissue LEV DNA Purification kit (Promega, Madison, WI, USA), which use magnetic resin beads. Each cell line samples was fixed with CytoRich Red solution and centrifuged at 1, 200 rpm and 4°C for 5 min, and the cell pellets were re-suspended and washed once with 95% ethanol. Next, the cells were re-pelleted by centrifugation at 1, 200 rpm and 4°C for 5 min and the pellets were air dried for 5 min at room temperature and processed using each DNA extraction kit according to the manufacturer’s instructions. After the DNA concentration was measured using the Qubit 3.0 Fluorometer dsDNA BR assay kit (Life Technologies), the DNA quality was confirmed using the QIAseq DNA quantimize kit (Qiagen). The extracted DNA was diluted to a concentration of 5–10 ng/μL for use as template DNA, PCR was performed using the QIAseq DNA quantimize kit and Ct values were calculated from 100- and 200-bp amplicons. In higher quality DNA, the Ct values from 100-bp (Ct100) and 200-bp (Ct200) amplicons should show lower variations; however, in lower quality DNA, the difference in the Ct values would be wider, indicating that the sample contains lower concentrations of amplifiable DNA fragments. Therefore, quality check (QC) score (ΔCt200 -ΔCt100 /200–100) was calculated to evaluate DNA quality, in which lower QC scores indicate reduced DNA degradation. The amplifiable DNA concentration (ng/μL) was calculated as 1/2^ΔCt150 × 5^, where ΔCt150 is the average of the ΔCt200 and ΔCt100 scores, and the number 5 represents the control amplifiable DNA concentration of 5 ng/μL.

### Preparation of DNA libraries from cultured human cancer cells and quality check

Capan-1, FTC133 and MCF-7 cells fixed in 5 mL CytoRich Red at a cell density of 6 × 10^3^ cells/mL were subjected to DNA extraction using the above-mentioned different methods after fixation for 90 days. For MCF-7 cells, the NGS library was constructed from DNA extracted by the four different kits using a panel of 93 breast cancer-related genes (QIAseq Human Breast Cancer Panel DHS-001Z, Qiagen). For Capan-1 and FTC133 cells, NGS libraries were generated using a panel of 160 cancer-related genes (GeneRead Comprehensive Cancer Panel, Qiagen) from genomic DNA extracted using three different extraction methods, excluding the Takara Bio kit. The quality of the NGS libraries was confirmed suing the Agilent high-sensitivity DNA kit (Agilent Technologies, Santa Clara, CA, USA) to confirm the successful generation of 300-bp products for the QIAseq Human Breast Cancer Panel and 160-bp products for the GeneRead Comprehensive Cancer Panel.

### Clinical cytology samples

Thirty-one residual LBC specimens collected from Kagoshima University Hospital, Japan, from 2014 to 2018, and six residual LBC samples from the Hokuto Hospital, Hokuto Social Medical Corporation in 2017, were used for the current study. The thirty-one samples from Kagoshima University Hospital were acquired during endoscopic retrograde cholangiopancreatography (ERCP). The six samples from Hokuto Hospital were taken by fine needle aspiration (FNA) from breast cancer tissues. The LBC specimens were fixed in approximately 5 mL CytoRich Red solution, and the residual samples were stored at 4°C for between 1 month and 4 years. The samples were processed for DNA extraction using the Maxwell Purification Kit, and DNA quality was confirmed as described above.

### Comprehensive cancer genome analysis for cultured cells and clinical cytology samples

After the cultured Capan-1, FTC133, and MCF-7 cells were fixed and stored in 5 mL of CytoRich Red at a density of 6 × 10^3^ cells/mL for 90 days at 4°C, DNA was extracted from the cells and used to construct NGS libraries using the GeneRead Comprehensive Cancer Panel. For MCF-7 cells, the QIAseq Human Breast Cancer Panel was also used to construct the NGS library. The above cancer panel has been clinically implemented as the PleSSision test (formerly known as the CLHURC test, Keio University PleSSision Group, Tokyo, Japan) [19]. For the LBC samples from breast cancer tissues obtained at Hokuto Hospital, an NGS library was constructed using the QIAseq Human Breast Cancer Panel. A total amount of 40–100 ng DNA for the QIAseq Human Breast Cancer Panel, and 10 ng DNA was used for the GeneRead Comprehensive Cancer Panel for library construction, and were applied to the MiSeq sequencer (Illumina, San Diego, CA, USA) after dilution with hybridization buffer to a final concentration of 20 pM. Finally, the sequencing data were analyzed by the Qiagen Web Portal service (https://www.qiagen.com/us/shop/genes-and-pathways/data-analysis-center-overview-page/).

### Statistical analyses

All values are expressed as the mean ± standard deviation. Significant differences were analyzed using Student’s *t*-test or one-way analysis of variance (ANOVA). Values of *p* < 0.05 were considered statistically significant.

### Ethical approval for the genome studies

The studies involving human genomic sequence from the culture cells were approved by the ethics committees for clinical and epidemiologic research at Kagoshima University. The studies using clinical LBC samples were approved by the ethics committees for clinical research at Hokuto Hospital, and written-informed consent was obtained from each participant.

## Results

### Cell density at fixation and recovery of DNA

**Fig 1:**
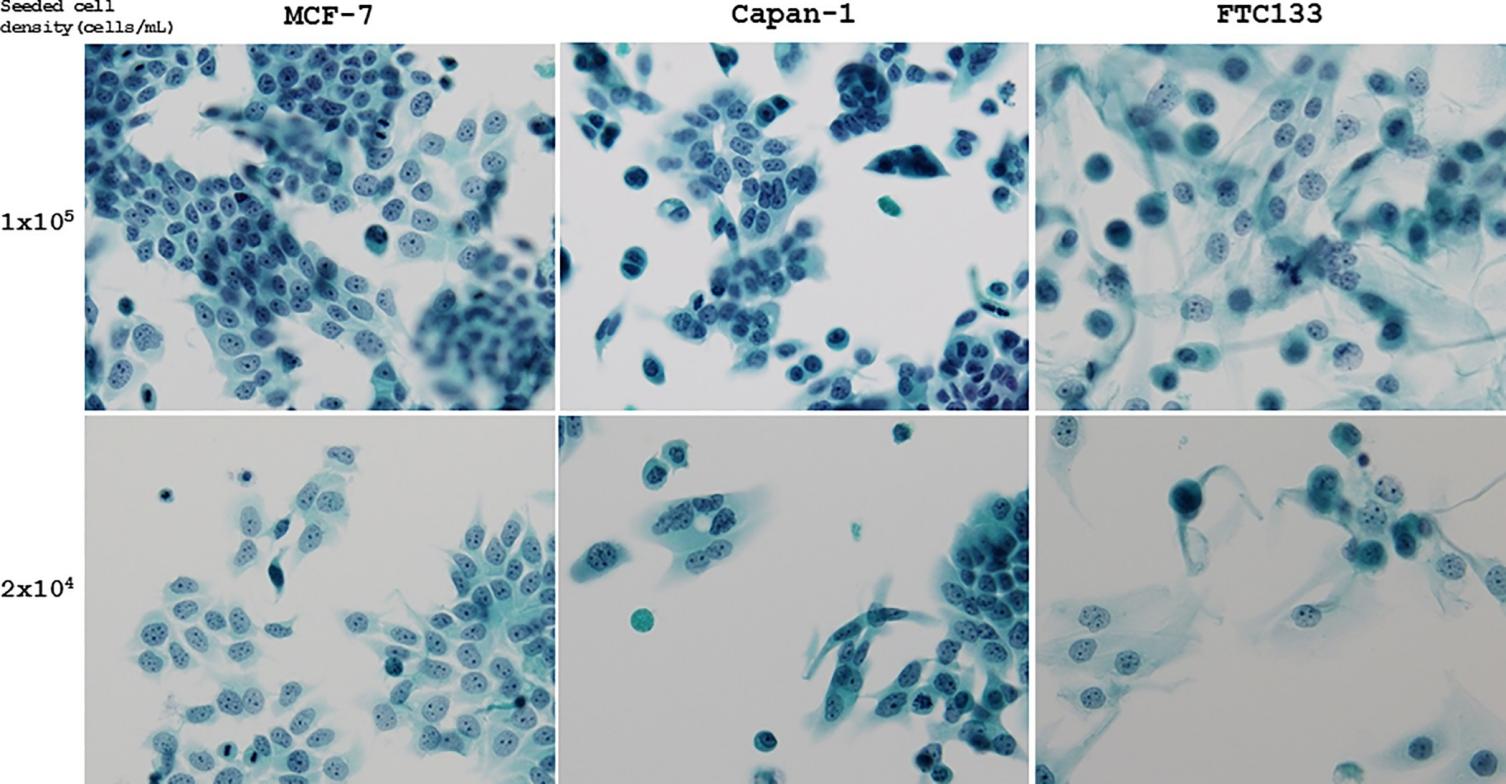
Representative LBC of cultured human cancer cells fixed at various cell densities. MCF-7, Capan-1, and FTC133 cells were stored for 90 days at 4°C after fixation with CytoRich Red solution. The cell morphology was well preserved and cell overlapping was less frequent at a density of 2 × 10^4^. In contrast, some cells overlapped at a cell density of 1 × 10^5^ cells/mL. (All cells were visualized by Papanicolaou staining, 200 × magnification).

**Fig 2:**
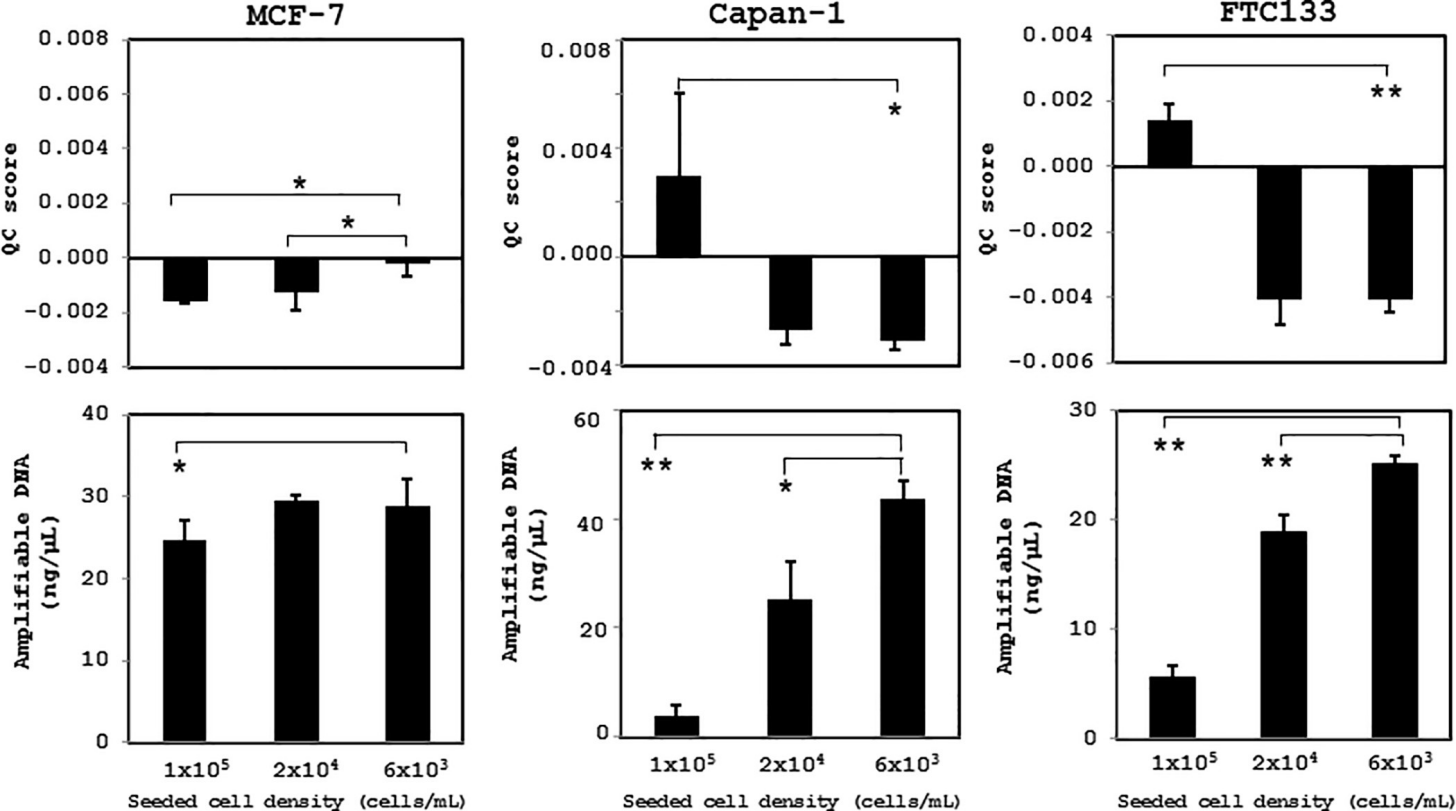
Quality of DNA extracted from cultured human cancer cells fixed at various cell densities. QC scores and amplifiable DNA concentrations were calculated in Capan-1, FTC133, and MCF-7 cells fixed at various cell densities in CytoRich Red solution for 1 week at 4°C. Lower cell densities of 6 × 10^3^ and 2 × 10^4^ cells/mL exhibited favorable QC scores and amplifiable DNA concentrations Maxwell 16 FFPE Tissue LEV DNA Purification kit. *, *p* < 0.05; **, *p* < 0.01.

The cultured cells were stored for 90 days at 4°C after fixation in CytoRich Red solution; the cell morphology was well preserved, and cell overlapping was less frequent at cell densities of 6 × 10^3^ and 2 × 10^4^ cells/mL than that of 1 × 10^5^ cells/mL. Many cells overlapped at a density of 1 × 10^5^ cells/mL, and it was sometimes difficult to observe the cell morphology for cytologic diagnosis (Fig 1). In all types of cultured cells stored for 1 week after fixation, a density at of 6 × 10^3^ or 2 × 10^4^ cells/mL was ideal to obtain the lowest QC scores and the highest amplifiable DNA concentrations using Maxwell 16 FFPE Tissue LEV DNA Purification kit (Promega) (Fig 2).

### DNA extraction methods and DNA quality over longer fixation time

**Fig 3:**
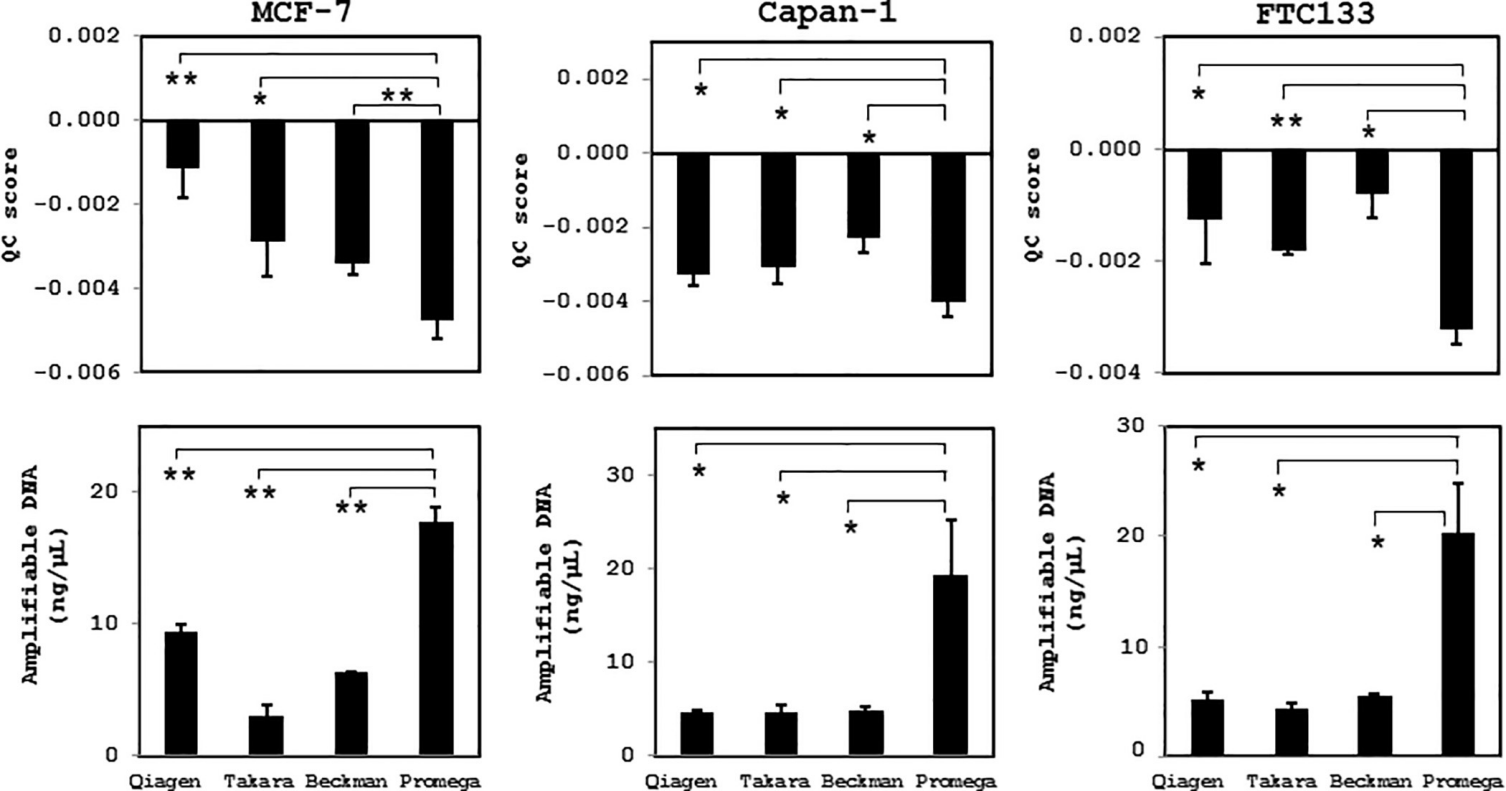
Quality of DNA extracted from cultured human cancer cells using different DNA extraction methods after fixation for 30 days. MCF-7, Capan-1, and FTC133 cells were fixed in CytoRich Red solution at a density of 6 × 10^3^ cells/mL for 1 month at 4°C, and then the quality of extracted DNA was evaluated based on QC scores and amplifiable DNA concentrations. The Maxwell 16 FFPE Tissue LEV DNA Purification kit (Promega) yielded the highest DNA quality. *, *p* < 0.05; **, *p* < 0.01. Qiagen, DNeasy Blood & Tissue kit; Takara, NucleoSpin Tissue XS kit; Beckman, Agencourt FormaPure DNA kit; Promega, Maxwell 16 FFPE Tissue LEV DNA Purification Kit.

**Fig 4:**
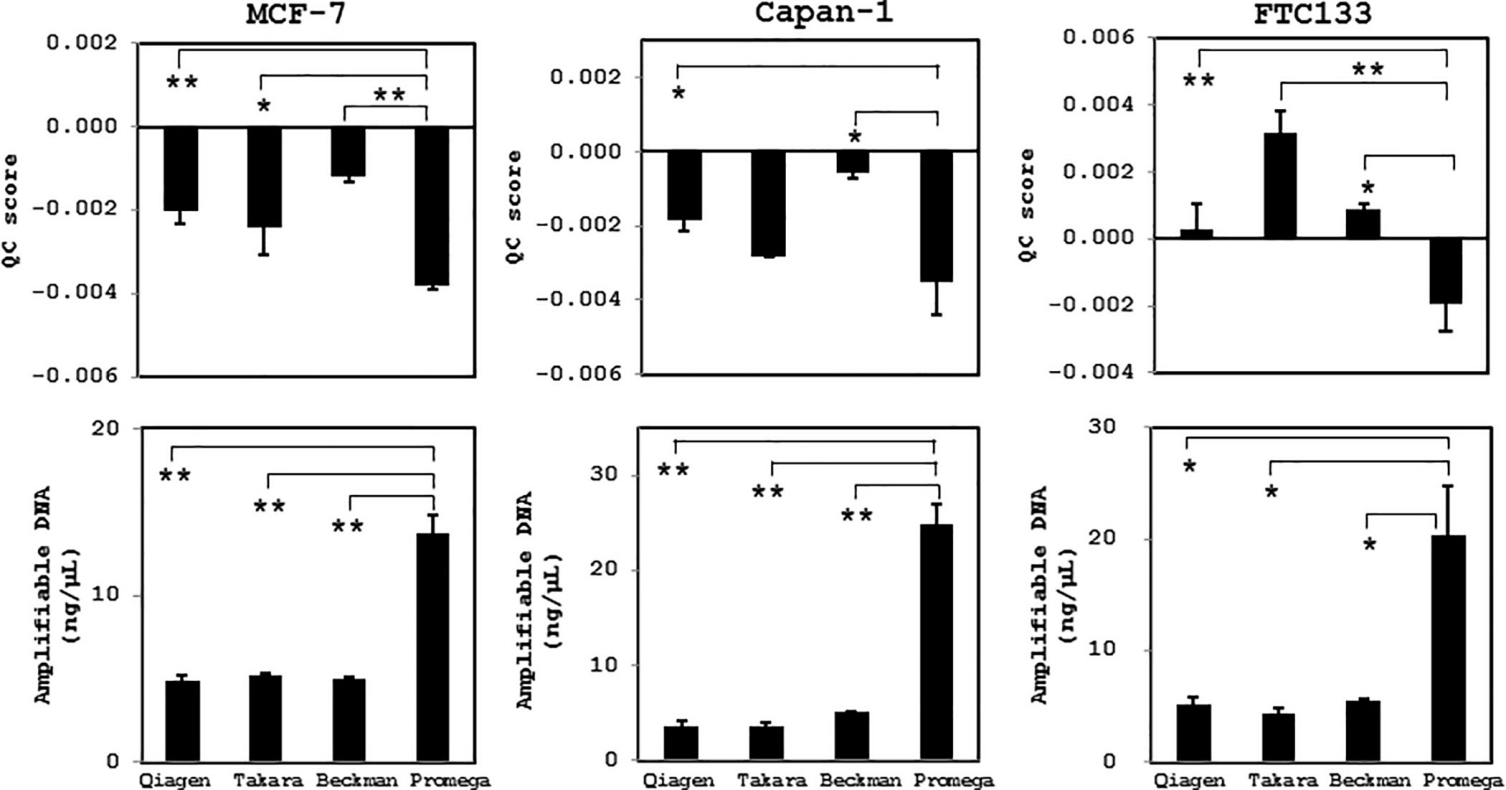
Quality of DNA extracted from cultured human cancer cells using different DNA extraction methods after fixation for 90 days. MCF-7, Capan-1, and FTC133 cells were fixed in CytoRich Red solution at a density of 6 × 10^3^ cells/mL for 3 months at 4°C, and then the quality of extracted DNA was evaluated based on the QC scores and amplifiable DNA concentrations. The Maxwell 16 FFPE Tissue LEV DNA Purification kit (Promega) yielded the highest DNA quality. *, *p* < 0.05; **, *p* < 0.01. Qiagen, DNeasy Blood & Tissue kit; Takara, NucleoSpin Tissue XS kit; Beckman, Agencourt FormaPure DNA kit; Promega, Maxwell 16 FFPE Tissue LEV DNA Purification Kit.

Next, we examined the variations in DNA quality obtained using the four different extraction methods from cultured cells at a density of 6 × 10^3^ cells/mL after 30 and 90 days of fixation. In all types of cell preparation, the magnetic resin beads-based extraction method using the Maxwell 16 FFPE Tissue LEV DNA Purification kit produced the highest quality of DNA, in which the lowest QC score and highest amplifiable DNA concentration were measured (Figs 3 and 4). Irrespective of the extraction method, however, the extracted DNA was of sufficient quality for NGS analysis (QC score cut-off value < 0.04) and did not display prominent DNA degradation.

### DNA extraction methods and NGS library quality

**Fig 5:**
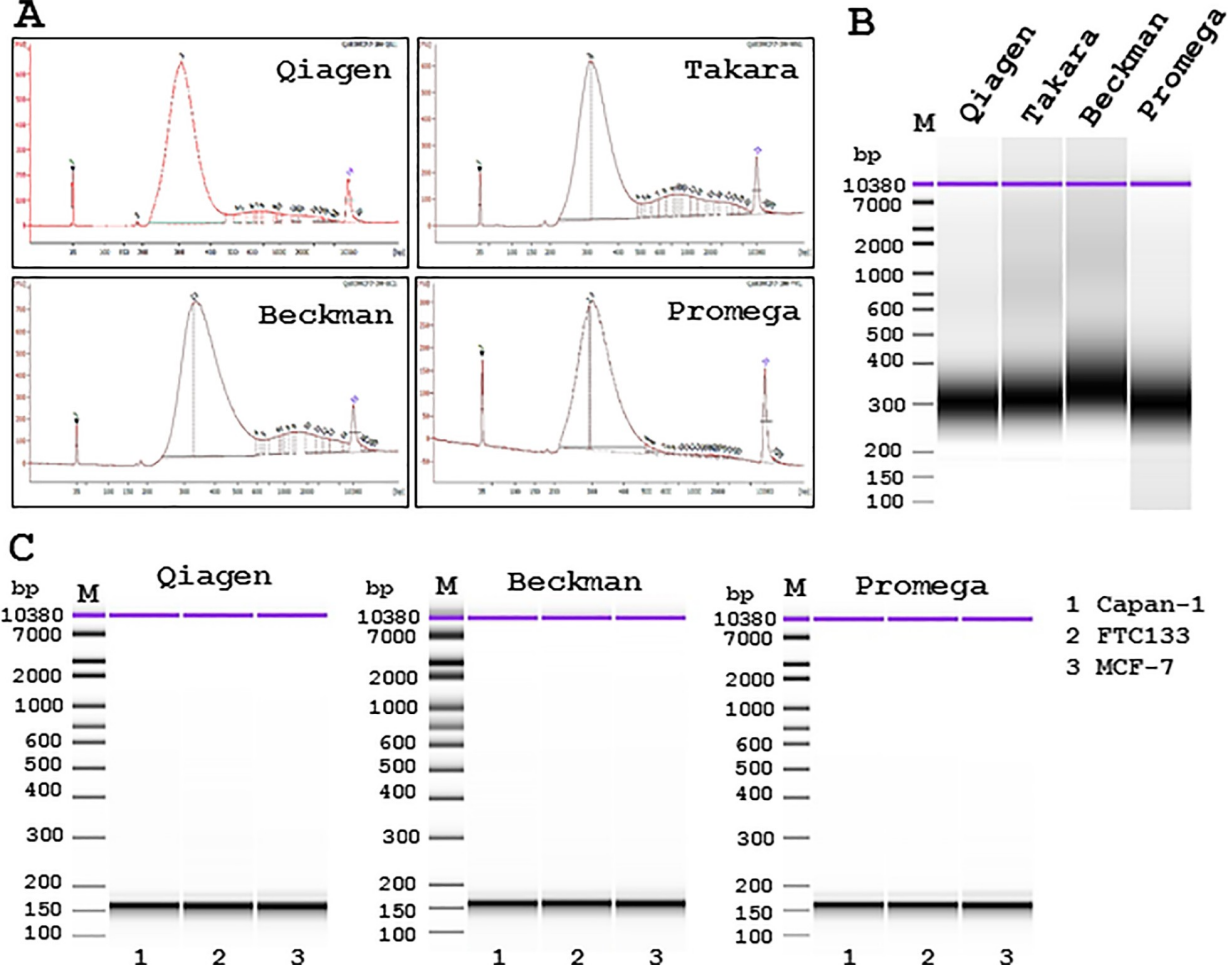
NGS library qualities of cultured human cancer cells with different DNA extraction methods. DNA was extracted from MCF-7 cells using four different extraction kits. The NGS library contained 300-bp PCR products using the QIAseq Human Breast Cancer Panel. The generation of 300-bp library PCR products was demonstrated using densitometry (**A**) and pseudo-electrophoresis imaging (**B**). Lane **1**, DNeasy Blood & Tissue kit (Qiagen); Lane **2**, NucleoSpin Tissue XS kit (Takara Bio); Lane **3**, Agencourt FormaPure DNA kit (Beckman Coulter) and Lane **4**, Maxwell 16 FFPE Tissue LEV DNA Purification Kit (Promega). Similar favorable results were obtained from cultured Capan-1, FTC133, and MCF-7 cells using three different extraction methods and the GeneRead Comprehensive Cancer Panel, which showed the generation of 160-bp library PCR products **(C)**. Lane **1**, Capan-1; Lane **2**, FTC133; Lane **3**, MCF-7 cells.

DNA was extracted from cultured cells using three or four different extraction kits and DNA prepared for NGS library construction. The library quality was then confirmed using the Agilent high sensitivity DNA kit. The library contained 300-bp PCR products for the QIAseq Human Breast Cancer Panel using DNA extracted from MCF-7 cells from any of the four different extraction methods (Fig 5A and 5B). With DNA extracted from Capan-1, FTC133, and MCF-7 cells, the libraries contained 160-bp products for the GeneRead Comprehensive Cancer Panel from three of the extraction methods (Fig 5C). All the libraries from all extraction methods were of sufficient quality for NGS analysis.

### DNA quality after long-term storage in CytoRich Red solution

**Fig 6:**
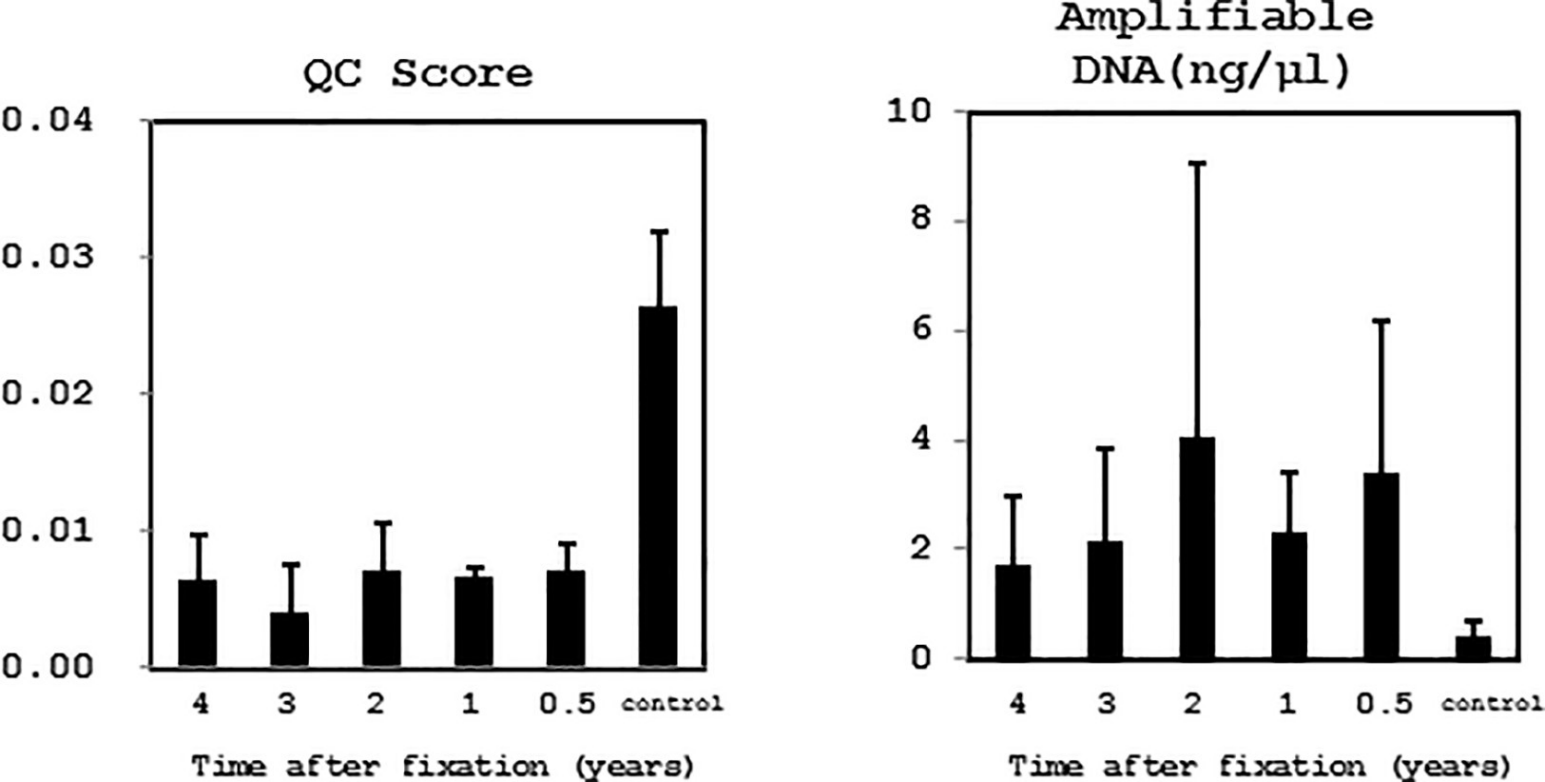
Quality of DNA extracted from clinical LBC samples after long-term storage in CytoRich Red solution. DNA was extracted using the Promega kit from LBC samples subjected to long-term storage at 4°C in CytoRich Red solution. In samples stored for 4 years, the QC scores gradually increased and amplifiable DNA concentration progressively decreased. However, even in these samples, the QC score less than the cut-off value of 0.04 and suitable for library construction. DNA extracted from FFPE tissues after storage for 4 years were used for as a control.

Residual LBC specimens obtained from the Kagoshima University Hospital were used to determine the effect of long-term storage at 4°C in CytoRich Red solution. DNA was extracted using the Promega kit. The QC score significantly increased, and the amplifiable DNA concentration decreased, in samples stored for 4 years, but the QC score was still less than 0.04 and applicable for library construction in all samples (Fig 6).

### Comprehensive genomic DNA analysis by NGS using cells fixed with CytoRich Red solution

DNA extracted from MCF-7 cells were subjected to NGS using the QIAseq Human Breast Cancer Panel with DNA extracted using the Promega kit, in which NGS results showed the same gene mutation variants as previously reported [20] (Table 1). The DNA extracted from Capan-1, FTC133, and MCF-7 cells were processed for NGS analysis using the GeneRead Comprehensive Cancer Panel. The NGS results, including mean read depth and variant allele fraction, were comparable, and variant calls were exactly same across the three different DNA extraction methods in each cell line (Table 2).

**Table 1:** NGS analysis of cultured human breast cancer MCF-7 cells.

DNAs extraction	QC score	Mean read depth	Variant call	VAF(%)	
**Promega**	**-0.004**	**472**	**GATA3p-355fs**	**50.6**	
			**PIK3CApGlu545Lys**	**53.4**	** **
**Beckman**	**-0.001**	**620**	**GATA3p-355fs**	**48.7**	
			**PIK3CApGlu545Lys**	**45.1**	** **
**Qiagen**	**-0.002**	**706**	**GATA3p-355fs**	**49.9**	
			**PIK3CApGlu545Lys**	**48.7**	** **
**Takara Bio**	**-0.002**	**576**	**GATA3p-355fs**	**47.2**	
			**PIK3CApGlu545Lys**	**52.7**	** **

VAF, Variant allele fraction; CNV, Copy number variation

**Table 2:** NGS analysis from cultured cancer cells.

cell	DNAs extraction	QC score	Mean read depth	Variant call	VAF(%)
**Capan-1**	**Promega**	**-0.003**	**557**	ARID1ApGln546*	**58.6**
				**KRASpGly12Asp**	**81.0**
				**TP53pArg273His**	**100**
	**Beckman**	**-0.001**	**825**	ARID1ApGln546*	**53.4**
				**KRASpGly12Asp**	**81.0**
				**TP53pArg273His**	**99.8**
	**Qiagen**	**-0.002**	**854**	ARID1ApGln546*	**49.8**
				**KRASpGly12Asp**	**79.9**
				**TP53pArg273His**	**99.5**
**FTC133**	**Promega**	**-0.002**	**625**	**ERBB4pVal748Leu**	**99.4**
				PTENpArg130*	**99.8**
				**TP53pArg273His**	**100**
				NF1pCys167*	**99.4**
				**ERBB2pAla830Val**	**99.5**
	**Beckman**	**0.001**	**947**	**ERBB4pVal748Leu**	**100**
				PTENpArg130*	**99.8**
				**TP53pArg273His**	**99.6**
				NF1pCys167*	**99.0**
				**ERBB2pAla830Val**	**99.7**
	**Qiagen**	**0**	**882**	**ERBB4pVal748Leu**	**98.9**
				PTENpArg130*	**99.9**
				**TP53pArg273His**	**99.9**
				NF1pCys167*	**100**
				**ERBB2pAla830Val**	**99.8**
**MCF-7**	**Promega**	**-0.004**	**705**	**GATA3p-335fs**	**48.0**
	**Beckman**	**-0.001**	**824**	**GATA3p-335fs**	**52.2**
	**Qiagen**	**-0.002**	**950**	**GATA3p-335fs**	**50.6**

VAF, Variant allele fraction

* indicates a generation of stop codon.

The residual LBC breast cancer samples collected from Hokuto Hospital that were initially obtained by FNA and fixed with CytoRich Red solution were subjected to DNA extraction using the Promega kit and processed for NGS using the QIAseq Human Breast Cancer Panel. Even 12-month-old samples exhibited favorable QC scores (< 0.04), and all six LBC samples stored for 1–12 months in CytoRich Red solution were successfully sequenced to identify gene variants and copy number variations (Table 3).

**Table 3:** NGS analysis from LBC samples for breast cancer FNA.

case	Intrinsic subtype	Storage time	QC score	Mean read depth	Variant call	VAF(%)	CNV (fold)	
**1**	**Luminal**	**12 months**	**0.004**	**466**	**GATA3pLeu397fs**	**36.6**	**none**	
**2**	**Luminal**	**8 months**	**0.002**	**435**			**PMS2 amp**	**(4.0)**
					**ESR1pVal422del**	**12.1**	**CCND1 amp**	**(5.4)**
					PTENpTyr176*	**38.5**	**MDM2 amp**	**(6.0)**
**3**	**TNBC**	**7 months**	**0.018**	**501**	**RETpPro140Arg**	**28.1**	**NBN amp**	**(4.2)**
							**CDKN2A loss**	**(0.98)**
							**CBFB loss**	**(1.0)**
							**CDH1 loss**	**(1.0)**
							**TP53 loss**	**(1.0)**
**4**	**Her2(+) DCIS**	**5 months**	**0.002**	**529**	**TP53pHis214Arg**	**21.2**	**PMS2 amp**	**(4.6)**
							**FN11 amp**	**(6.7)**
							**ERBB2 amp**	**(6.9)**
							**CHEK2 amp**	**(4.0)**
**5**	**ER(+) SpCC**	**4 months**	**0.005**	**392**	**AKT1pGlu17Lys**	**44.9**	**none**	
					**TP53pArg280Lys**	**23.0**		
**6**	**TNBC**	**1 month**	**0.001**	**418**			**PTGFR loss**	**(0.7)**
							**NEK2 amp**	**(5.2)**
							**FBXO32 amp**	**(4.3)**
							**MYC amp**	**(5.2)**
					**PIK3CApGlu542Lys**	**60.5**	**CDKN2A amp**	**(6.2)**
					**TP53pPro278His**	**74.7**	**GATA3 amp**	**(11.5)**
							**AKT1 amp**	**(4.5)**
							**RAD51 amp**	**(4.7)**
							**CBFB amp**	**(6.6)**

TNBC, Triple negative breast cancer; Her2, Human epidermal grwoth factor receptor 2; DCIS, Ductal carcinoma in situ; ER, Estrogen receptor; ER(+)SpCC, ER positive spindle cell carcinoma; VAF, Variant allele fraction; CNV, Copy number variation

* indicated a generation of stop codon.

## Discussion

In the present study, we clearly demonstrated that DNA obtained from cultured cells and clinical LBC specimens fixed with CytoRich Red solution were suitable for cancer genome analysis using NGS, even after the samples were stored for up to 1 year. The DNA quality of DNA extracted using four different DNA extraction methods was comparable, and all the samples were suitable for the construction of NGS libraries and for cancer genome analysis.

A cell density of 6 × 10^3^ cells/mL for fixation and magnetic bead-based DNA extraction using the Promega kit, with any fixation time, resulted in the highest quality and quantity of DNA; the DNA was also of suitable quality for light microscopic LBC observation. A higher density of 1 × 10^5^ cells/mL resulted in a reduced concentration of amplifiable DNA, indicating that the DNA extraction kits displayed certain use limitations in our experimental conditions. However, the DNA quality was high enough for NGS when DNA was extracted using the other magnetic resin-based DNA extraction kit (Agencourt FormaPure DNA kit), although this kit showed lower extraction efficiency than the Promega purification kit. This might be due to the different nature of the magnetic resins. In terms of whole exome sequences from FFPE tissues, DNA extracted using the QIAamp DNA FFPE tissue kit and GeneRead DNA FFPE kit (both are membrane-based extraction kits from Qiagen) previously demonstrated higher DNA quality (in terms of DNA integrity), a higher variant calling frequency, and higher coverage of genes of interest than those extracted using the Promega purification kit [21]. It is possible that the differences in variant calling and gene coverage, attributed to the different extraction methods, were not detected in our study owing to the small number of target genes analyzed. Alternatively, it is possible that these previous results were not comparable to ours because we used cultured cells fixed with an alcohol-based fixative, rather than FFPE. The selection of the DNA extraction method according to the types of samples appears to be essential for the quality of genomic DNA.

In addition to the DNA extraction method, another factor influencing the DNA quantity and quality is the fixative agent. Although formaldehyde degrades DNA during the long-term storage of FFPE tissue [22, 23], the CytoRich Red fixative, which consists of 20–25% isopropanol, 10% methanol, 5–10% ethylene glycol, and 1% formaldehyde, did not result in marked DNA degradation in the present study. This reduced formaldehyde concentration for LBC preparation did not promote DNA degradation and did not inhibit PCR in our NGS.

Dejmek et al. [24] reported that the yield of DNA is affected by how cultured cells are processed and placed on glass slides for cytology, and that CytoLyt fixative (Hologic, Marlborough, MA, USA) showed a five-fold increased DNA recovery compared to CytoRich Red solution. In terms of the DNA quality, however, they did not perform a comparative study between CytoLyt fixative and CytoRich Red solution [24]. Instead, PCR amplification was compared between DNA extracted from conventional smear slides (May-Giemsa staining after air drying, and Papanicolaou staining after isopropanol-based spray fixation) and those extracted from cells fixed with CytoRich Red solution. The results showed that a 760-bp PCR product for the *EGFR* exon 20 was not detected in the air-dried samples, and showed inconclusive amplification from CytoRich Red-fixed cells; in contrast, PCR products were clearly detected using the Papanicolaou slides. When LBC specimens were fixed with CytoRich Red and DNA was extracted from the cells on glass slides, Kim et al. similarly failed to detect *BRAF* exon 15 (a 226-bp PCR product) [25]. Another study reported poor or no detection of *EGFR* exon 19 (a 190-bp PCR product) from DNA extracted from cultured cells fixed in CytoRich Red fixative for more than 1 day [26]. The experimental protocol of this previous study was very similar to that of the present study, except that they used a higher cell density of 1 × 10^6^ cells/mL for fixation. In our study, the cultured cells were fixed at a much lower density of 6 × 10^3^, 2 × 10^4^, or 1 × 10^5^ cells/mL. We demonstrated favorable DNA quality and satisfactory NGS results from the cells fixed at lower density of 6 × 10^3^ cells/mL.

On the other hand, DNA extracted using the QIAamp DNA Micro kit (Qiagen) from cultured PC9 and PC11-18 cells and fixed with CytoRich Red in a previous study were a successful source of DNA for the PCR amplification of *EGFR* mutations [27]. Satisfactory results of the PCR amplification of *KRAS* mutations were also achieved using a fluorescence resonance energy transfer-based preferential homoduplex formation assay in CytoRich Red-fixed cells directly obtained from endoscopic ultrasound-guided FNA and the QIAamp DNA FFPE Tissue kit for DNA extraction [28]. Furthermore, the effective multiplex PCR analysis for NGS using a lung cancer hot-spot panel has been reported, where DNA was extracted directly from clinical LBC samples using the Gentra Puregene DNA extraction kit (Qiagen) [29]. In that previous study, the cells obtained by endobronchial ultrasound-guided transbronchial needle aspiration were fixed with CytoLyt and preserved in PreservCyt fluid (Hologic). In the present study, the extracted DNA from residual LBC samples fixed with CytoRich Red solution; ERCP and FNA, showed high quality for NGS analysis, and the latter samples were successfully applied for comprehensive breast cancer genome analysis. It is assumed that clinically obtained LBC specimens would display lower cellularity than experimentally prepared samples from cultured cells. Therefore, the cell density at fixation in any alcohol-based fixatives and the source of DNA—directly from LBC samples or from cells on glass slides—rather than the method of DNA extraction, might be critical for determining DNA quality and its suitability for NGS.

The LBC samples were shown to be suitable for NGS analysis when DNA was directly obtained from LBC samples fixed with the appropriate concentration of fixative. In fact, NGS analysis of thyroid gland needle aspiration/biology specimens has already been incorporated as a routine strategy for the determination of appropriate therapeutic strategy for thyroid follicular tumors including goiters, follicular adenomas and carcinomas [30–32]. Since cytology is a less invasive and less expensive diagnostic procedure, further increasing the availability of LBC samples for comprehensive cancer genome analysis would promote precision medicine.

## Supporting information

S1 File Supporting information for Figs 2–6. (ZIP)

S2 File Cell NGS data for Tables 1 and 2. (ZIP)
